# Atomistic Insights into Anomeric and Stereochemical Effects on Glucose Transport by GLUTs

**DOI:** 10.1021/jacs.5c17290

**Published:** 2026-02-02

**Authors:** Brian Wiley, Leonardo Cirqueira, Richard J. Naftalin, Carmen Domene

**Affiliations:** † Department of Chemistry, 1555 University of Bath, 1 South Building, Claverton Down, Bath BA2 7AX, United Kingdom; ‡ BHF Centre of Research Excellence, School of Medicine and Life Sciences, 150716 King’s College London, Waterloo Campus Stamford St, London SE1 9HN, United Kingdom

## Abstract

Although β-glucose is more abundant than α-glucose in aqueous solution, GLUT3 preferentially binds α-glucose due to favorable interactions and conformational complementarity within the protein binding site. This study explores the anomeric preferences of glucose transporters GLUT1 and GLUT3 for α- and β-glucose using classical MD, providing mechanistic insight into previously reported differences in anomer-specific transport rates during net influx, efflux, and exchange flux, as well as asymmetric binding of glucose anomer derivatives. Analysis of hydrogen-bonding frequencies between glucose anomers and transporter residues, combined with root mean squared fluctuations (RMSF) of these residues during flooding simulations, using either mixed α/β-glucose trajectories or single-anomer trajectories, reveals distinct residue preferences along the transport pathway. GLUT3 residues exposed to the extracellular solution preferentially interact with α-d-glucose, while inward-facing residues show a bias toward β-d-glucose. This distributed network of anomer-selective interactions, particularly concentrated in extramembranous surface regions, highlights previously unrecognized complexity in GLUT stereoselectivity. Enhanced residue displacements adjacent to orthosteric glucose collision sites suggest that allosteric intra- and interchain interactions may contribute to the cooperative transport behavior observed in mixed α + β-glucose simulations compared to single-anomer conditions. Importantly, anomeric stereoselectivity in GLUT1 and GLUT3 is not confined to the central high-affinity binding site, but also involves a multiplicity of extramembranous residues, underscoring the broader structural basis for selective glucose transport.

## Graphical abstract

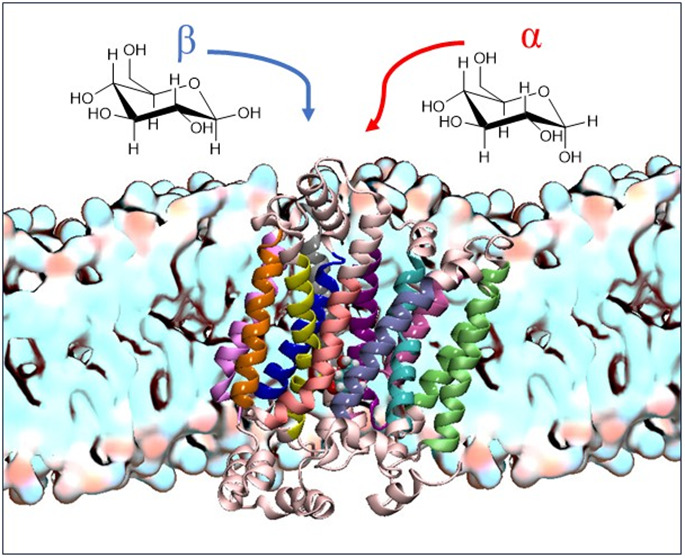

## Introduction

Glucose exists in solution as interconverting α- and β-D-anomers (Figure 1). At equilibrium in aqueous solutions, the β-anomer, with an equatorial C1 hydroxyl group, predominates at 62% while the α-anomer, with an axial C1 hydroxyl group, constitutes 38% of the content.^1^ The intermediate open chain form exists in negligible concentrations, typically less than 0.25%.^2,3^ The hydration behavior of these anomers differ significantly; β-d-glucose forms more hydrogen bonds with surrounding water molecules and exhibits shorter average sugar–water hydrogen bond lengths compared to α-d-glucose.^1^ These characteristics result in more symmetrical hydration shells around β-d-glucose, contributing to its higher solubility in water. These structural differences influence how glycans are recognized by lectins and enzymes. The geometric and physicochemical compatibility between a glycan and its binding site is affected by the anomeric configuration, which can alter binding affinity and specificity. While hydrogen bonding and stereoelectronic effects are primary drivers of glycan recognition, hydrophobic interactions may also contribute by stabilizing specific glycan conformations within nonpolar regions of the binding site.^4–6^ For clarity and in accordance with common usage, we omit the D prefix in subsequent references to these anomers.

**Figure 1:**
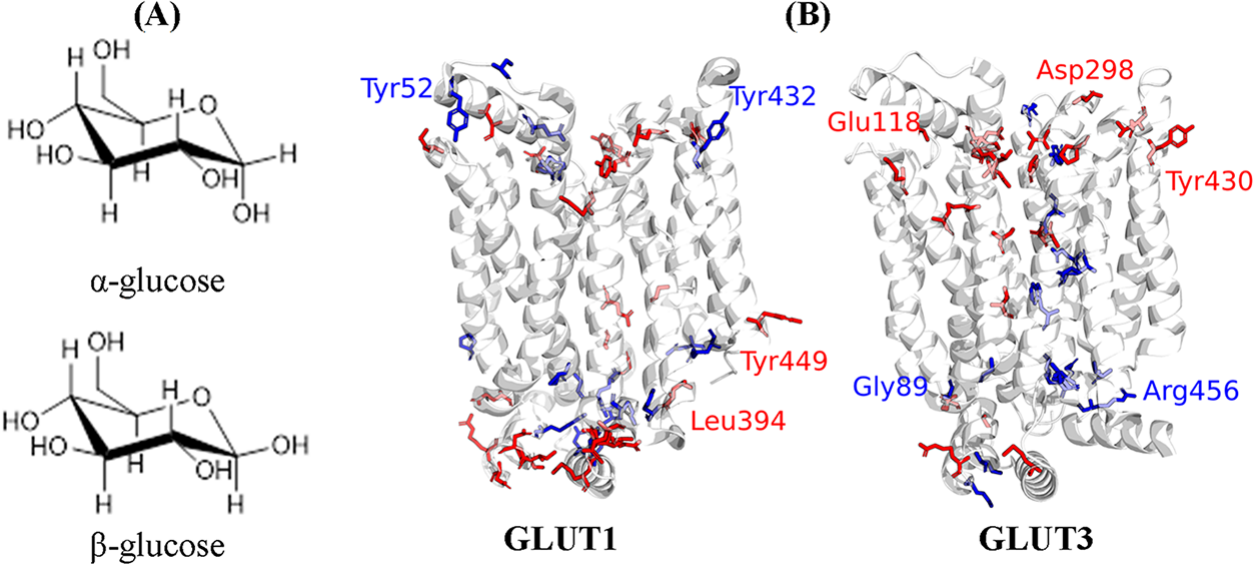
(A) Chemical structures of α- and β-glucose. (B) Map of anomeric preferences of GLUT1 and GLUT3 residues, as presented in Table 3. GLUT1 does not exhibit significant anomeric preference on either its extracellular or intracellular surface, whereas GLUT3 shows a markedly higher α-glucose preference on the extracellular surface compared to the intracellular surface. Red indicates α-glucose preference, blue indicates β-glucose preference. Some residues are labeled for orientation and reference. Residues with no clear preference are provided in the Supporting Information.

Research spanning six decades has investigated whether glucose transporters (GLUTs) exhibit preferences for specific glucose anomers. Despite extensive study, results remain inconsistent across different methodologies. Table 1 summarizes key studies on anomer preferences, highlighting the methodological variations that may contribute to differing results. Studies using C1-fluoro glucose anomers, which do not anomerize, suggest that the β-anomer binds more strongly to GLUT1 ^7^ than the α-isomer and is transported more rapidly.^8,9^ However, other conflicting evidence suggests α-anomer preference, particularly at low substrate concentrations. The transport rate ratio, apparent K_m_/apparent *V*_max_, is faster for the α-anomer.^10^ Furthermore, GLUT3 crystal structures show greater abundance of α-glucose, despite β-glucose predominating in the surrounding solution, suggesting that the α anomer is preferred.^11^ Interpretation of these latter experiments is complicated by rapid interconversion of α- and β-glucose anomers and by hetero exchanges between the anomers (αβ and βα exchanges).^12^

**Table 1:** Summary of Published Research on Anomeric Transport via GLUT1^a^

citation	*T* (°C)	concentration (mM)	β/α ratio	method
Faust^8^	37	300	3.70	Net inflow Glu
			1.03	Net inflow D_2_O Glu
Ehwald et al.^13^	35	67	0.32	*S. Cerevisiae* net uptake rate
Miwa et al.^14^	37	3.0	1.72	Pancreatic islets
Kuchel et al.^1^	40	25.5	0.60	Human RBC efflux
Potts, Kuchel.^15^	37	50–60	0.87 ± 0.07	Exchange α and ß 3FG
Potts et al.^16^	37	9.3	2.15	3FG exchange
Carruthers and Melchior^17^	0.6	20	1.05	Net uptake Glu.
	36.6		1.03	Net uptake Glu.
Fujii et al.^18^	25	16.7	1.20 *p* < 0.001	Net uptake at 5s
Naftalin and Rist^19^	24	24	1.0	D_2_O substitution does not affect 3-OMG exchange in rat RBCs
Duan et al.^2^	8	12.5	1.24	rRat RBC metabolized uptake
Janoshazi and Solomon^20^	9.3	5	α → β-glucose	GLUT1 Trp quenching
O’Connell et al.^21^	37	10	0.93	Efflux 2F-Glu
			0.77	Efflux 3F-Glu
			0.39	Efflux 4F-Glu
London and Gabel^9^	37	10	Pb > Pa	2F-Glu exchange hRBC
Barnett et al.^7^	25	10	0.18	Kt β: α ratio Glu 1F
Leitch and Carruthers^22^	4	100	1.0	Exchange Glu, 3-OMG
Bresciani, et al.^23^	37	10	0.22	Efflux Glu exch. in D_2_O
			0.73	Efflux 3F Glu exch. in D_2_O
Dickinson et al.^24^	37	5	0.96	Efflux of 3F Glu in D_2_O
Shishmarev et al.^10^	37	6.2	0.94	FDG-2
		24.8	0.76	FDG-2
			0.59	Efflux 3F Glu in D_2_O

a Reported experimental conditions include temperature (*T*), substrate concentration, and observed transport preference expressed as the β/α anomer ratio. Methods used in each study are indicated, allowing comparison of experimental approaches and outcomes across citations.

Several factors are likely to contribute to the inconsistent results observed across the previously published transport studies in GLUT1, shown in Table 1. The first is that the β/α anomeric preference differs significantly between net glucose influx studies, net glucose efflux studies, and exchange transport. At low concentrations, the α-anomer typically shows a higher transport coefficient than the β-anomer.^10^ A second factor is fluorine substitution for glucose hydroxyl groups. Although this is useful for NMR studies, it affects glucose’s physicochemical properties. Fluorination generally increases hydrophobicity by reducing polarity and solvation, although the extent depends on the substitution pattern. Mono- and difluorinated glucose derivatives often exhibit increased affinity for certain enzymes, attributed to enhanced hydrophobic interactions and altered electronic properties.^25^

Solvent effects are another contributing factor: substituting D_2_O for H_2_O while clarifying NMR signals, also affects net β-glucose flux more than α-glucose flux but has a minimal impact on exchange transport.^8,19^ This differential effect of D_2_O may relate to β-glucose’s greater hydrophilicity, as the more symmetrical hydration shells surrounding β-glucose may be more sensitive to changes in solvent properties.^4–6^ Finally, differences in transport mechanisms play a role. Net transport and exchange transport operate through distinct mechanisms: net flux depends primarily on gating mechanisms at both ends of the transporter’s central pore, whereas exchange occurs through simultaneous ligand exchanges within intramolecular cavities and only when these cavities contain multiple interacting ligands.^26^ Glucose exchange processes are much less temperature-sensitive than net flux, as they occur within the fluid-filled intramolecular cavities, while the gating processes controlling net flux are influenced by temperature-sensitive movements of the aromatic side chains of the bottleneck residues and salt-bridge interactions within the extramembranous zones.^26^ These latter processes are subject to compressive forces exerted by the membrane lipids, which increase following temperature-sensitive fluid-to-gel phase transitions.^27^

The differing mechanisms between the exchange and net transport modes could account for the observed differences between anomer preferences for exchange and net glucose transport and the differential anomer preferences for exchange at high and low ligand concentrations (Table 1).^10^ Glucose exchange involves more ligand contacts with intramembranous residues than net flux, and features a more uniform distribution of interactions throughout the transporter. In contrast, net flux is characterized by an asymmetric distribution of ligands across the membrane, with a predominant bias toward interactions with cis-facing residues.

Crystallographic studies of GLUT3 reveal that hydrophobic residues surround the bound hexose ring, stabilizing it through van der Waals interactions. Notably, α-glucose is more frequently observed in GLUT3 crystal structures, despite β-glucose being the predominant anomer in aqueous solution. However, these structures primarily capture high-affinity binding sites and may overlook lower-affinity or transient interaction sites that are relevant under physiological conditions. Yan’s group has highlighted the key role of the hydrophobic residue Trp388 in GLUT1, which contributes to substrate binding and conformational transitions.^11,28^ In these crystallographic studies, ligand uptake was achieved by prolonged coincubation in the crystallization buffer. This mode of glucose access, while effective for structural resolution, does not reflect the physiological mechanism of substrate entry when the transporter is embedded in its native membrane environment.

Molecular dynamics (MD) trajectories of α- and β-glucopyranose in GLUT1 and GLUT3 provide detailed insights into the anomeric preferences of individual residues and residue groups within the transporters. The current simulations were performed using systems containing either individual anomers or equimolar mixtures of α- and β-glucopyranose, resulting in separate trajectories for each condition. By comparing the frequency and duration of glucose–residue interactions across these trajectories, we identified residues that participate in cooperative anomer interactions. This analysis focuses on hydrogen bonds that occur at either higher or lower proportions of the total bond count when both anomers are present together, relative to when only a single anomer is present.

Understanding whether GLUT transporters exhibit anomeric selectivity is not only central to resolving decades of conflicting biochemical observations but also critical for appreciating their physiological roles. GLUT1 and GLUT3 represent two of the most important glucose transporters in human biology: GLUT1 governs basal glucose uptake in erythrocytes and the blood–brain barrier, while GLUT3 ensures high-affinity glucose transport in neurons. Even subtle preferences for α- or β-glucose could shift intracellular glucose dynamics, influencing enzymatic flux, glycosylation pathways, and neuronal energy metabolism. Because glucose anomers interconvert slowly in solution, small transporter biases may exert disproportionate effects under physiological conditions. Although glucose anomers interconvert spontaneously in aqueous solution, the rate of mutarotation is relatively slow, on the order of tens of minutes at neutral pH, compared with the millisecond-to-second time scales of enzymatic or transport processes. In bulk solution, such as blood plasma, this rate may be sufficient to maintain equilibrium between α- and β-glucose over minutes, but during rapid transport events the spontaneous interconversion is too slow to sustain equilibrium. The presence of mutarotase in certain tissues, such as the kidney, further indicates that spontaneous mutarotation alone is not always adequate to meet physiological demands. Consequently, even a modest transporter preference for one anomer could have a disproportionate impact on glucose availability, as slow interconversion would amplify the kinetic effects of anomeric selectivity. The mutarotase activity of human erythrocytes has been observed to be negligible.^21^ Moreover, clarifying anomer recognition has implications for drug design, the development of glucose-based imaging tracers, and the interpretation of experiments using fluorinated analogues. By systematically investigating GLUT1 and GLUT3 with both structural and computational approaches, this study aims to resolve inconsistencies in the literature and provide mechanistic insight into how anomeric configuration shapes glucose transport across membranes.

## Experimental Procedures

### Materials and Methods

To investigate the anomeric preferences of GLUT1 and GLUT3, molecular dynamics simulations were setup using experimentally resolved crystal structures embedded in model membrane environments.

### System Set-Up

The crystal structure of the human glucose transporter GLUT1 (PDB ID: 4PYP), which has a nonyl β-d-glucopyranoside bound, was used as a starting point for the computational work.^29^ The initial systems were generated using the Membrane Builder module of CHARMM-GUI.^30^ A membrane patch of 100 Å × 100 Å dimensions was built. The membrane contained 205 molecules of 1, 2-dipalmitoyl-*sn*-glycero-3-phosphocholine (DPPC). This choice was based on a compromise between size and computational resources. Subsequently, the GLUT1 structure was inserted into the membrane patch. To avoid steric clashes, lipids in close contact with the protein were deleted. The combined system was solvated with water and ions up to a final concentration of 150 mM NaCl to produce a rectangular simulation box of dimensions 95 × 95 × 105 Å^3^ and ∼100,000 atoms.

Crystal structures of GLUT3 were retrieved from the Protein Data Bank, specifically, the outward-occluded conformation (PDB 4ZW9, 1.5 Å resolution).^11^ Monoolein molecules were removed from the crystal structures, while sugar molecules were either retained in the original positions or removed depending on the simulation setup. Default protonation states were assigned to ionizable residues, supported by PropKa calculations.^31^ SOLVATE1.0 was used to solvate the protein and fill cavities. A pre-equilibrated DPPC lipid bilayer was used, and the protein was aligned to the bilayer normal before insertion into the membrane. Lipid molecules within 1.2 Å of protein atoms were deleted. Sodium and chloride ions were added to neutralize the system and achieve a biologically relevant ion concentration (150 mM), using the Autoionize plugin in VMD.^32^ Water molecules overlapping with protein, lipids, or ions (distance < 1.2 Å) were removed, resulting in a final system size of approximately 80,000 atoms.

### Molecular Dynamics Simulations

Simulations were conducted using a flooding approach, in which the system was exposed to high concentrations of glucose, allowing the substrate to partition freely into the membrane and the embedded transporter throughout the molecular dynamics trajectory. All degrees of freedom of the protein were left unrestrained, resulting in a fully flexible and dynamic system that included explicit water molecules and a realistic membrane lipid environment. This setup also permitted intramolecular interactions between glucose molecules, enabling the identification of multiply occupied binding sites within the transporter.

The MD simulations were performed with NAMD 2.13 software.^33^ The CHARMM36 force field^35^ was used to model the protein, glucose, and lipids. Standard CHARMM parameters were used for ions,^34^ and the TIP3P model was applied for water.^35^ Pressure was maintained at 1 atm using a Langevin piston,^36^ with a damping time constant of 50 ps and a period of 200 ps. A semi-isotropic pressure coupling method was used in all the simulations. For the NAMD calculations, the piston pressure acted independently in each dimension but maintained a constant ratio in the *x* and *y* axes, corresponding to the plane of the membrane. The temperature was kept constant at 323.15 K by coupling the system to a Langevin thermostat,^37^ with a damping coefficient of 1 ps^–1^. The particle mesh Ewald (PME) algorithm^38^ was used for the evaluation of electrostatic interactions beyond 12 Å, with a PME grid spacing of 1 Å, and NAMD defaults for spline and κ values. A 12 Å cutoff was applied to nonbonded forces. Electrostatic and van der Waals forces were smoothly switched off between 10 Å and 12 Å using the default NAMD switching function. A Verlet neighbor list with pair-list distance of 13.5 Å was used to evaluate nonbonded neighboring forces within the pair-list distance.^40^ The lengths of covalent bonds involving hydrogen atoms were constrained using the Shake algorithm to use a 2 fs time-step.^39^ The multitime step algorithm Verlet-I/r-RESPA was used to integrate the equations of motion.^40,41^ The systems were subjected to 10,000 steps of energy minimization, followed by equilibration involving the sequential release of various restraints added to the system: (i) harmonic restraints to heavy atoms of the protein and ions, (ii) repulsive restraints to prevent water from entering in the hydrophobic region of the membrane, and (iii) planar restraints to maintain the position of the lipid headgroups along the *z*-axis. Subsequently, production runs were executed at the selected temperatures. The systems underwent 10,000 steps of minimization and equilibrated for a total of 3.0 ns. The duration of each equilibration step was 1 ns with a gradual reduction of restraints and constraints (1) protein atoms, lipid headgroups, and sugars with a force constant of 20 kcal/mol Å^2^ for 0.5 ns and a force constant of 10 kcal/mol Å^2^ and 5 kcal/mol Å^2^ for 0.25 ns each; (2) protein atoms with a constraint scaling of 5 kcal/mol Å^2^ during 1 ns; and (3) backbone atoms with a constraint scaling of 5 kcal/mol Å^2^ during 1 ns. A summary of the simulations is provided in Table 2.

**Table 2:** Summary of Simulations Performed^a^

system notation	anomer	# replicas/simulation time [μs]	# glucose molecules
GLUT1 β-glucose	β	1/2.1	48
GLUT1 α/β-glucose	α + β	3/1.0	24 + 24
GLUT3 α-glucose	α	1/6.0	60
GLUT3 β-glucose	β	1/4.8	60
GLUT3 α/β-glucose	α + β	3/1.0	30 + 30

a MD simulations considered in this study and some system details.

## Results

The relative frequencies of glucose anomer interactions with GLUT are used here as a direct method for comparing anomeric preferences, with residues presented in Table 3 and Figure 1. These simulations were conducted using both separate trajectories of individual anomers and equimolar mixtures of α- and β-glucopyranoses. By comparing the frequency and duration of glucose anomer hydrogen bonding to each residue across different simulation conditions, it is possible to establish which residues participate in cooperative anomer interactions. The analysis focuses on glucose-residue hydrogen bonds that occur at either higher or lower frequencies in the presence of both anomers, compared to simulations with only a single anomer (Figure 2).

**Figure 2:**
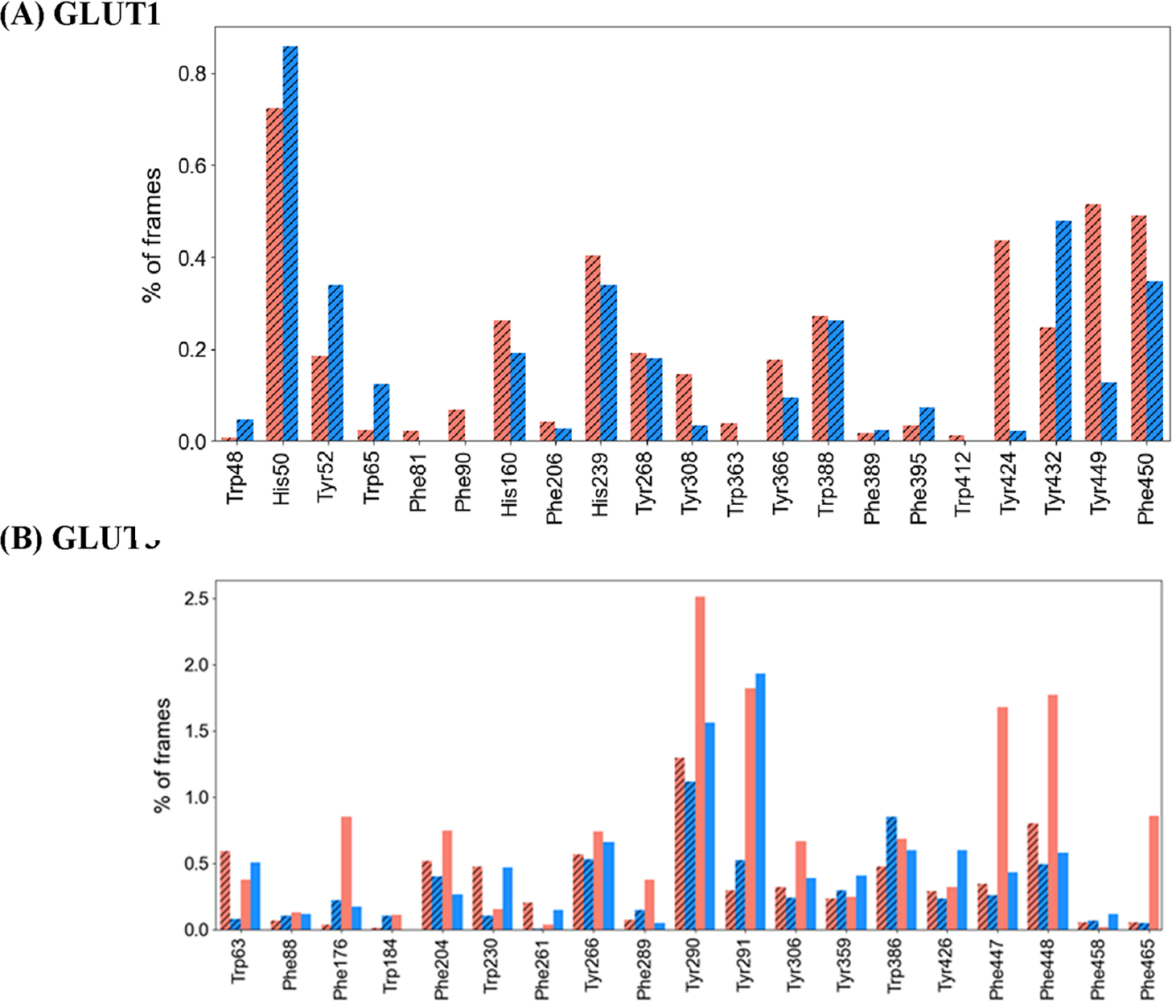
Hydrogen-bonding preferences of glucose anomers, α-glucose (red) and β-glucose (blue), from MD simulations. Plots show hydrogen-bond frequencies from mixture simulations for GLUT1 (A), and single and mixed-anomer simulations for GLUT3 (B). Hashed columns represent the proportions observed under equimolar α:β conditions. For **GLUT3**, (B) the largest reductions in β-glucose H-bonding in mixtures occur at Trp63, Trp230, Tyr291, and Tyr426. Additional anomer-selective residues include Trp184, Phe261, Phe289, Tyr290, Tyr426, Phe447, Phe448, and Phe458, with preferences varying between single-anomer and mixed simulations. For **GLUT1**, (A) β-glucose shows higher H-bonding at Trp48, His50, Tyr52, Trp65, Phe395, and Tyr432, whereas α-glucose is favored at Phe90, His160, His239, Tyr308, Trp363, Tyr366, Tyr424, Tyr449, and Phe450.

**Table 3:**
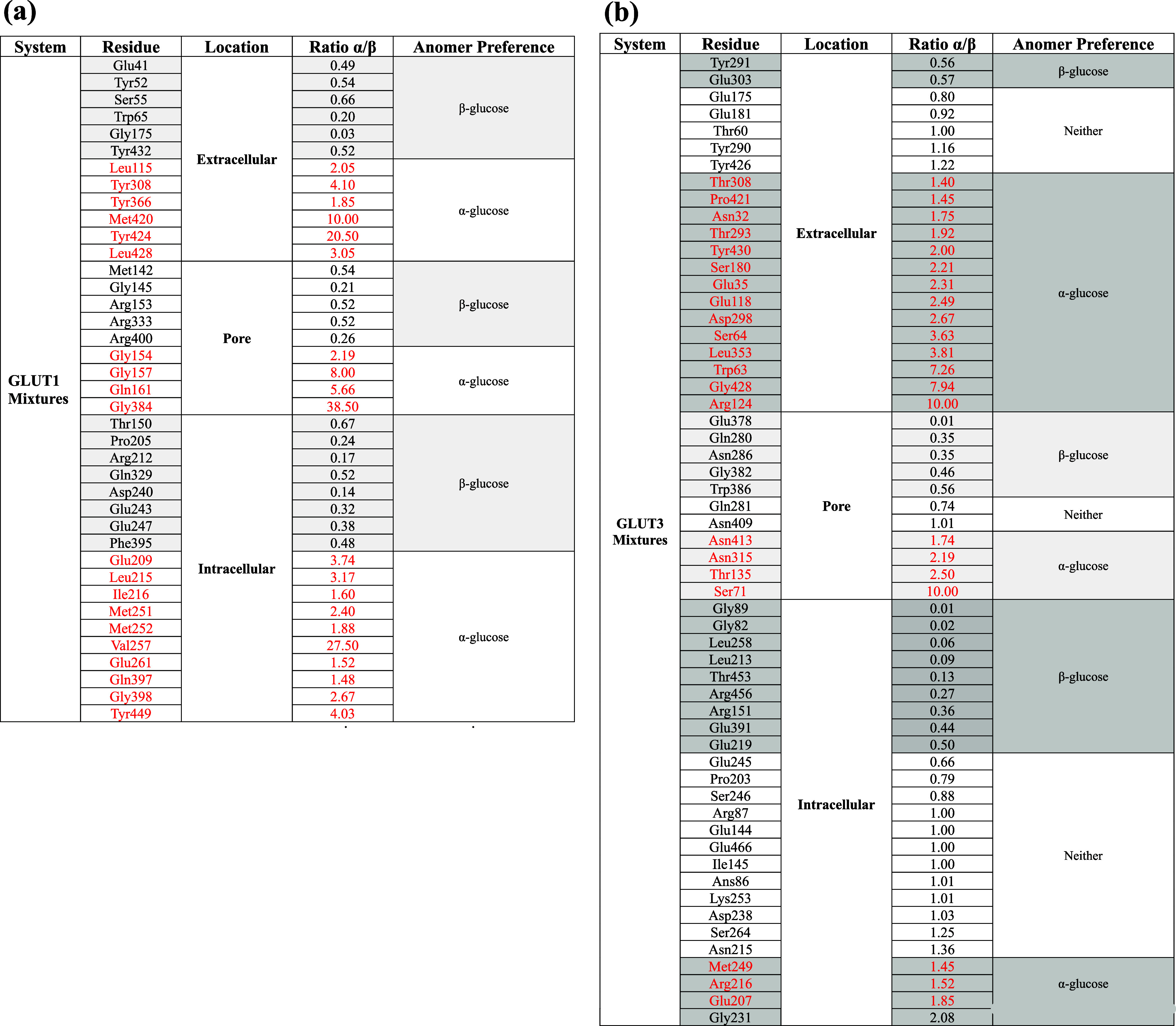
(A) Ratios of Glucose Hydrogen Bonds, Defined as the Proportion of α-Glucose to β-Glucose Interactions with Residues in GLUT1 Derived from Mixed-Anomer Trajectories^a^; (B) Ratios of Glucose Hydrogen Bonds, Defined as the Proportion of α-Glucose to β-Glucose Interactions with Residues in GLUT3, Derived from Mixed-Anomer Trajectories^a^

a Anomeric preference was assigned using the following thresholds: ratios ≥ 1.4 indicate α-preference, and ratios ≤ 0.7 indicate β-preference.

GLUT1 does not exhibit strong anomeric preferences between its external and internal surface residues (Figure 3 and Table 3), and only a slight bias is observed: in the outer extramembranous zone, six residues exhibit α-preference and six exhibit β-preference; in the inner extramembranous zone, eight residues show β-preference and ten show α-preference. In comparison to GLUT3, the pore region of GLUT1 displays no significant anomeric bias, with five residues showing α-preference and four showing β-preference. Preferences were defined using the following thresholds: a ratio ≥1.4 indicates α-preference, a ratio <0.7 indicates β-preference, and values between these thresholds indicate no clear preference. While the α/β ratio in the GLUT1 pore does not differ significantly from that of its external or internal surfaces, it is significantly higher than the α/β ratio observed in the GLUT3 pore region (Table 3).

**Figure 3:**
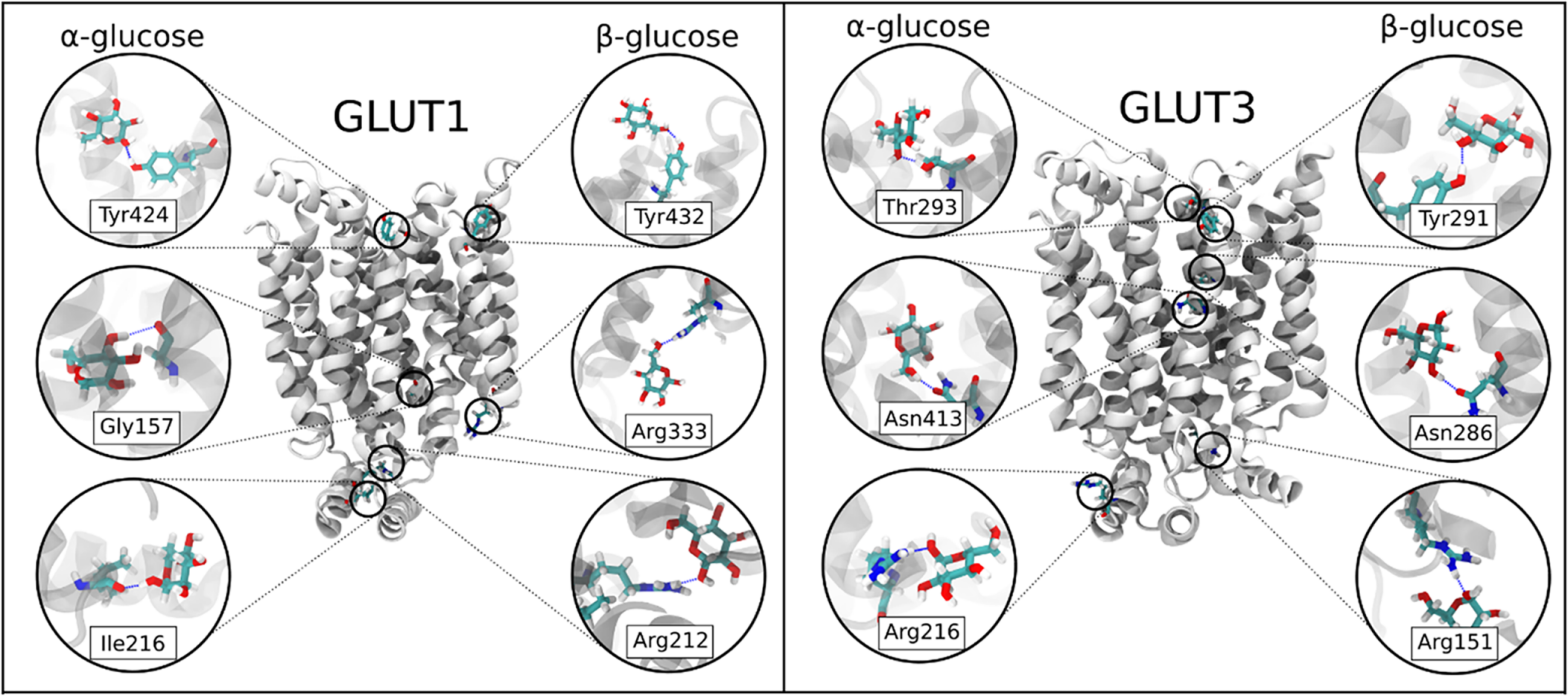
Structural visualization of hydrogen-bonding interactions between glucose anomers and key residues in GLUT1 and GLUT3 identified from the simulations. The protein is shown in cartoon representation (white), while the glucose anomer (α or β) and the interacting residues are shown in licorice representation. Panels show representative hydrogen-bonding interactions for α- and β-glucose in the extracellular vestibule, central cavity (pore region), and intracellular vestibule. For each region, two representative interactions reported in Table 3 are shown as examples for both GLUT1 and GLUT3.

When exposed to mixed anomer solutions, most GLUT3 residues facing the external solution show greater sensitivity to hydrogen bonding with α-glucose than with β-glucose (Table 3). Specifically, 14 external residues show α-preference, two show β-preference, and five exhibit no preference. In contrast, on the internal surface, four residues show α-preference, nine show β-preference, and 12 have no clear anomeric preference.

Under mixed anomer exposure, within the GLUT3 pore region, four residues prefer the α-anomer, five prefer the β-anomer, and two show no preference. The same criteria for defining preference (ratios ≥ 1.4 for α, < 0.7 for β, and intermediate values as neutral) were applied.

These findings are consistent with previous investigations of GLUT1 anomer influx, efflux, and inhibition, which demonstrated an α-glucose preference for hydrophobic glucose derivatives at the external surface and a β-glucose preference for the hydrophilic residues on the inward-facing surface of the transporter.^7,9,42^ Currently, there are no comparable in vitro transport studies investigating the anomer preferences of the neurally expressed GLUT3.

In the central channel of GLUT3, which serves as the preferred route for sugar transit, the constituent residues exhibit nearly equal affinities for the two anomers: four residues show a preference for α-glucose, three favor the β-anomer, and four display no clear preference. Residues that favor α-glucose when exposed to an equimolar mixture of both anomers likely reflect the intrinsic in vitro anomeric preference more accurately, as they retain selectivity despite competition from the β-anomer. By comparison, the central channel of GLUT1 exhibits a higher overall preference for the α-anomer, with four residues favoring α-glucose, two preferring the β-anomer, and one showing neutral preference. Notably, in both GLUT3 and GLUT1, one of the principal exit routes from the central pore to the intracellular solution is formed by an annulus of residues enriched in β-anomer preference.

We note, however, that residue-level statistics within the pore should be interpreted with caution, as glucose entry into the central pore is a rare event in our simulations (see Figure 1 in the Supporting Information), limiting the statistical robustness of anomer-specific contact frequencies.

The trajectories reveal that the stereospecific preferences of glucose anomers are not simply confined to a few residues located at the central ‘high-affinity’ site of the transport channel^42^ but are distributed more widely within both inward and outward-facing vestibules and extramembranous regions. This more extensive distribution indicates that GLUT specificity is considerably more complex than previously assumed.

Additionally, the effects of anomer interactions with GLUTs on residue fluctuations (RMSFs) were analyzed to assess the broader impact of localized glucose interactions on the surrounding microenvironment. The propagation of these orthosteric collisions to adjacent residues through local interactions provides insight into how allosteric effects may be transmitted across the transporter structure.

The all-residue root mean squared fluctuations (RMSFs) provides a useful way of demonstrating anomer-specific effects. Ligand collisions with protein residues generate atomic displacements registered as fluctuations of the spatial positions of the protein Cα atoms. The fluctuations in extramembranous zones are much larger, as collision velocities of solution ligands and water molecules are ≈ 10 times greater than those occurring within the intramolecular regions and the protein chains are less constrained by the lateral forces exerted by the lipid membrane.^27^ The current study compares the RMSFs of transporter trajectories exposed to flooded conditions with either single α- or β-anomer, or equimolar concentrations of both anomers in the bathing solutions (Table 4).

**Table 4:** Single-Anomer vs Mixture Simulations^a^

	α-glucose (mixed simulation)	β-glucose (mixed simulation)
GLUT1	Asp236, Thr238, His239, Asp240, Gln242, Glu243, Met244, Lys245, Glu246, Glu247, Ser248, Met251, Ala392, Glu393, Thr455	Asn34, Ala35, Gln37, Pro58, Thr59, Arg218, Glu254, Lys255, Asn288, Tyr292, Thr295, Gln427, Cys429
GLUT3	Asn51, Gly82, Leu83, Gly380, His425	Leu46, Thr47, Gly50, Gln251, Lys253, Gly294, Lys297, Asp298, Ala299, Gly300, Val301, Gln302

	α-glucose (single-anomer simulation)	β-glucose (single-anomer simulation)
GLUT3	Ile177, Leu178, Gly179, Ser180, Glu181, Gln254, Glu327, Arg328, Ala329, Gly330, Gly382, Pro383	Leu46, Thr47, Gly231, Thr232, Ser236, Gln237, Asp238, Gln240, Glu241, Asp44, Glu245, Ser246, Ala247, Aarg248, Mer249, Gln251, Gln302

a Listed are residues involved in α- and β-glucose interactions identified from single-anomer and mixed simulations, based on heatmaps of RMSFs averaged over 10 × 25 ns windows. The first two rows correspond to the mixed GLUT1 and GLUT3 simulations shown in Figure 4, and the final row corresponds to the single α- vs β-glucose simulations for GLUT3 shown in Figure 6.

While RMSF representations of GLUT1 and GLUT3 in Figure 4 map the residue fluctuations averaged over the trajectory duration, they cannot be correlated with short-term transient events. By reducing the RMSF sampling periodicity to 25 ns and incorporating the trajectory time on the *x*-axis, while displaying fluctuation amplitudes in pseudocolor on the *y*-axis, a more precise and better delineation of events occurring in regions of interest, such as transient bonding interactions and residue fluctuations, can be obtained, as shown in Figure 4.

**Figure 4:**
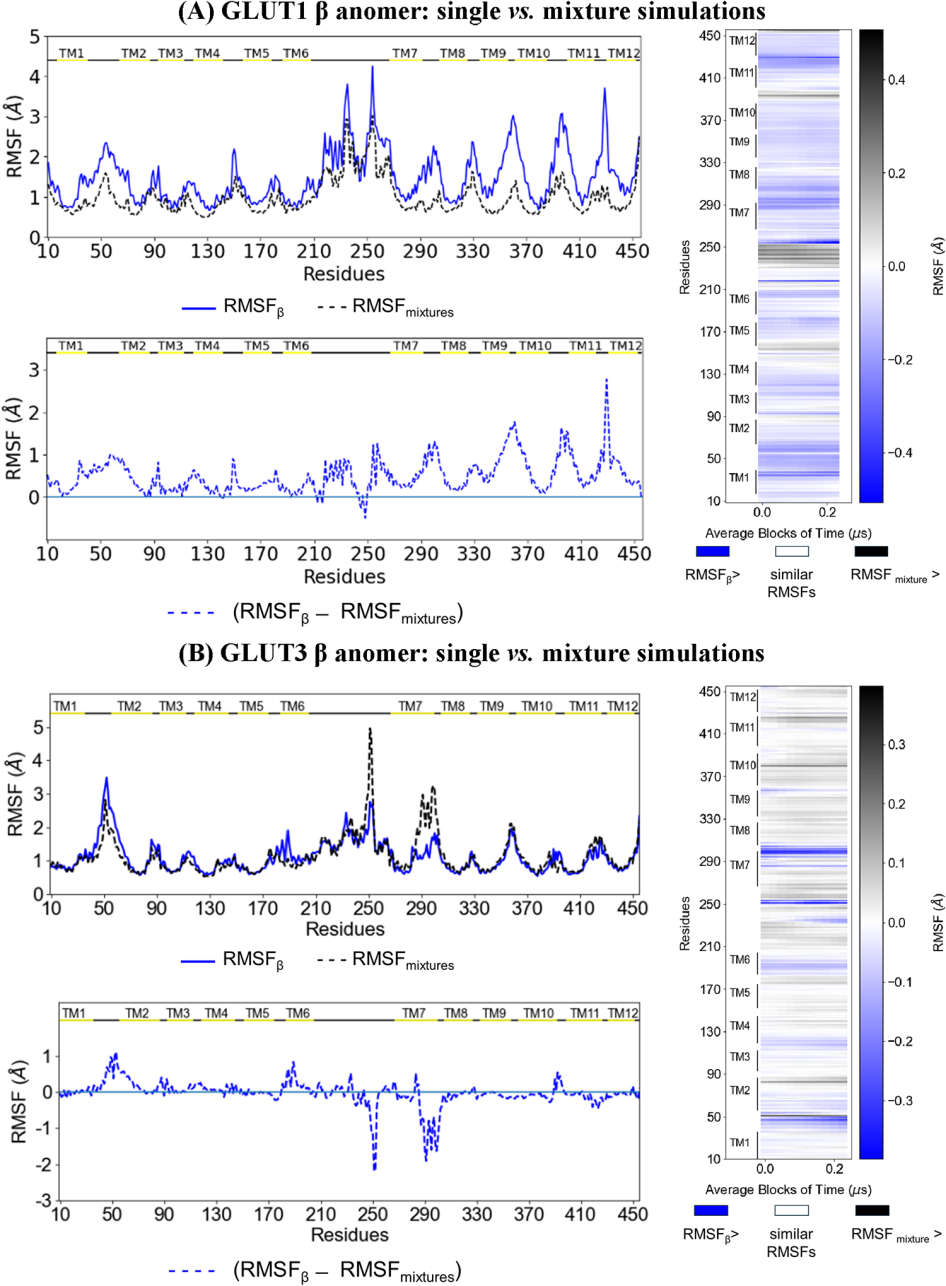
(A) GLUT1 exposed to flooding with either β-glucose (solid blue line) or a mixture of both anomers (averaged over three replicates; 24 molecules of each; dotted black line). For GLUT1 only, the RMSF values from single-anomer β-glucose simulations are higher than those from mixed-anomer simulations, especially in transmembrane helices (TMs) 2, 4, 6, 10, and 11, as well as the linkers between various TMs and the C-terminus. The heatmap shows the difference in RMSF between β-glucose and mixed-anomer simulations, averaged over 250 ns intervals from the 1-μs trajectories. Black denotes regions with higher RMSF in the mixed-anomer simulations, averaged across three replicates, whereas blue denotes regions with higher RMSF in the β-glucose single-anomer simulations. Notably, the mixed-anomer simulations transiently perturb intracellular (IC) residues between TM6 and TM7. (B) GLUT3 exposed to flooding with either β-glucose (solid blue line) or a mixture of both anomers (averaged over three replicates; 30 molecules of each; dotted black line). As in (A), the heatmap represents the RMSF difference between β-glucose and mixed-anomer simulations, averaged over 250 ns intervals across 1-μs trajectories. The mixed-anomer simulations perturb residues in TMs 2, 10, and 11, as well as various residues between TM6 and TM7, though to a lesser extent than in GLUT1. β-glucose perturbs the linker between TM1 and TM2, Gln251, Lys253, and residues at the top of TM7 above the Gly284 hinge over short periods. The color code is the same as in (A).

By selecting events coinciding with ligand entry into or retreat from extramembranous zones and occurring close to salt bridges or channel bottlenecks, the study identified localized residues that consistently responded with increased fluctuations to one or the other glucose anomer. The results reflect the observed preference for hydrophobic glucose derivatives at the external surface and a greater affinity for more hydrophilic derivatives at the inward-facing transporter surface.

The RMSF signals from single-anomer simulations are larger than those from mixed-anomer simulations in GLUT1. The larger fluctuations typically occur within clusters of three to five residues rather than isolated residues, and α > β, as shown in Figure 4. The current results illustrate that residues in GLUT1 and GLUT3 exhibit anomeric stereospecificity with a much broader distribution than previously recognized, being present within both inward- and outward-facing regions (Table 4).

From flooding simulations of GLUT1 and GLUT3 in the presence of both α- and β-glucose, the trajectories of individual glucose molecules were tracked both along the central pore of the transporter and in the surrounding aqueous environment, allowing visualization of their pathways relative to the entire protein surface (Figure 5 and Supporting Information Figures S2, and S3). The simulations were referenced to the crystal structure of GLUT3 (PDB 4ZW9), providing a structural frame for interpreting the observed glucose routes. Only glucose molecules that remained within the pore or interacted with extramembranous residues for longer than 4 ns were included in the analysis, thereby excluding over 95% of short-lived interactions (<2 ns) with the outer vestibular regions. For visualization purposes, α-glucose and β-glucose were color-coded red and blue, respectively, and the *z*-axis was oriented relative to the center of mass of the lipid membrane. This allowed a clear spatial understanding of the glucose movements relative to the membrane plane.

**Figure 5:**
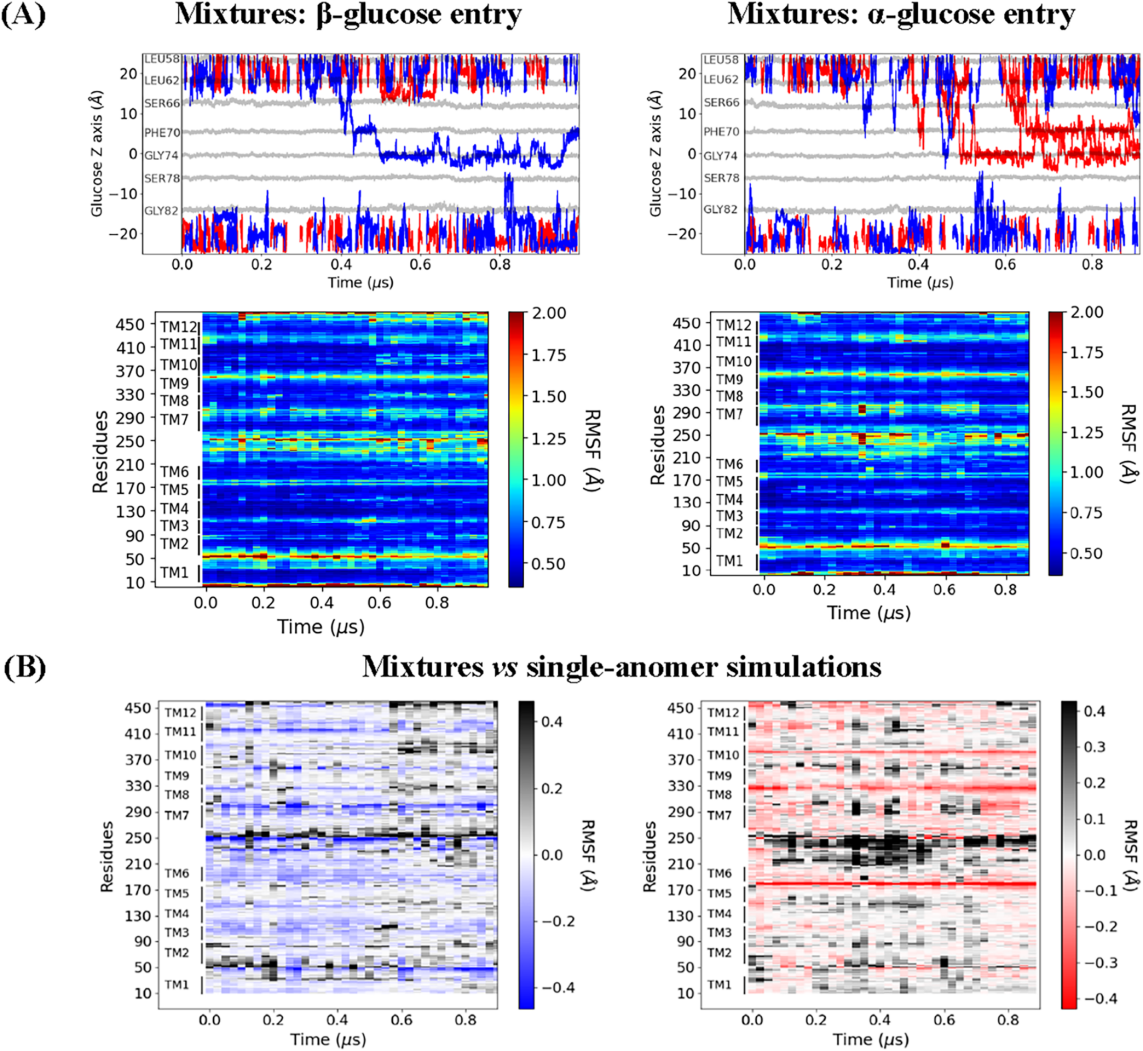
Comparisons of RMSFs in GLUT3. (A) Top: Glucose pathways through the pore from mixture simulations (PDB 4ZW9); only molecules in the pore or interacting with extramembranous residues for >4 ns are shown (α: red, β: blue). *Z*-axis origin is set at the membrane center. Bottom: Time evolution of Cα displacements relative to the starting structure for two representative mixture simulations. (B) Sliding-window comparisons of long single-anomer vs mixture simulations. Left: RMSF difference between the mixture replicate at the time β-glucose enters the pore (∼1 μs) and the individual β-glucose simulation (∼5 μs). Right: RMSF difference between the mixture replicate at the time α-glucose enters the pore (∼1 μs) and the individual α-glucose simulation (∼6 μs).

The results indicate that the rate-limiting steps of glucose transport are not restricted to the central high-affinity binding site. Residues at the outer and inner boundaries of the pore, including salt bridges and constriction points at either end of the central channel, act as stereospecific gating structures. These residues are located within linker chains that span extramembranous and vestibular zones, and they appear to play a key role in discriminating between glucose anomers. Previous studies have shown that these peripheral sites can mediate stereospecific interactions, and our simulations further confirm the presence of anomer-sensitive gating mechanisms beyond the canonical binding site.

A critical element of stereoselectivity is the ability of these sites to detect small conformational differences between ligands. Anomerization provides a convenient way to probe this selectivity, as residues can respond to variations in the orientation around the anomeric carbon, its attached hydroxyl group, the neighboring hydrogen, and the overall symmetry differences between asymmetrical α-glucose and the more symmetrical β-glucose.

The effects of anomer-specific interactions are not limited to directly bonded residues. Differential responses can propagate through neighboring residues, altering their fluctuations and dynamics within the local 3D environment. Sliding-window analyses of root-mean-square fluctuations were used to quantify these effects, with windows of 910 ns for single-anomer simulations (∼5–6 μs total) and 250 ns for comparisons of α- vs β-glucose entering the pore in mixture simulations (Figure 5B). These analyses reveal that certain residues move more in individual anomer simulations (indicated by red shading), while others move more in mixture simulations (blue shading), reflecting complex, context-dependent modulation of protein dynamics.

**Figure 6:**
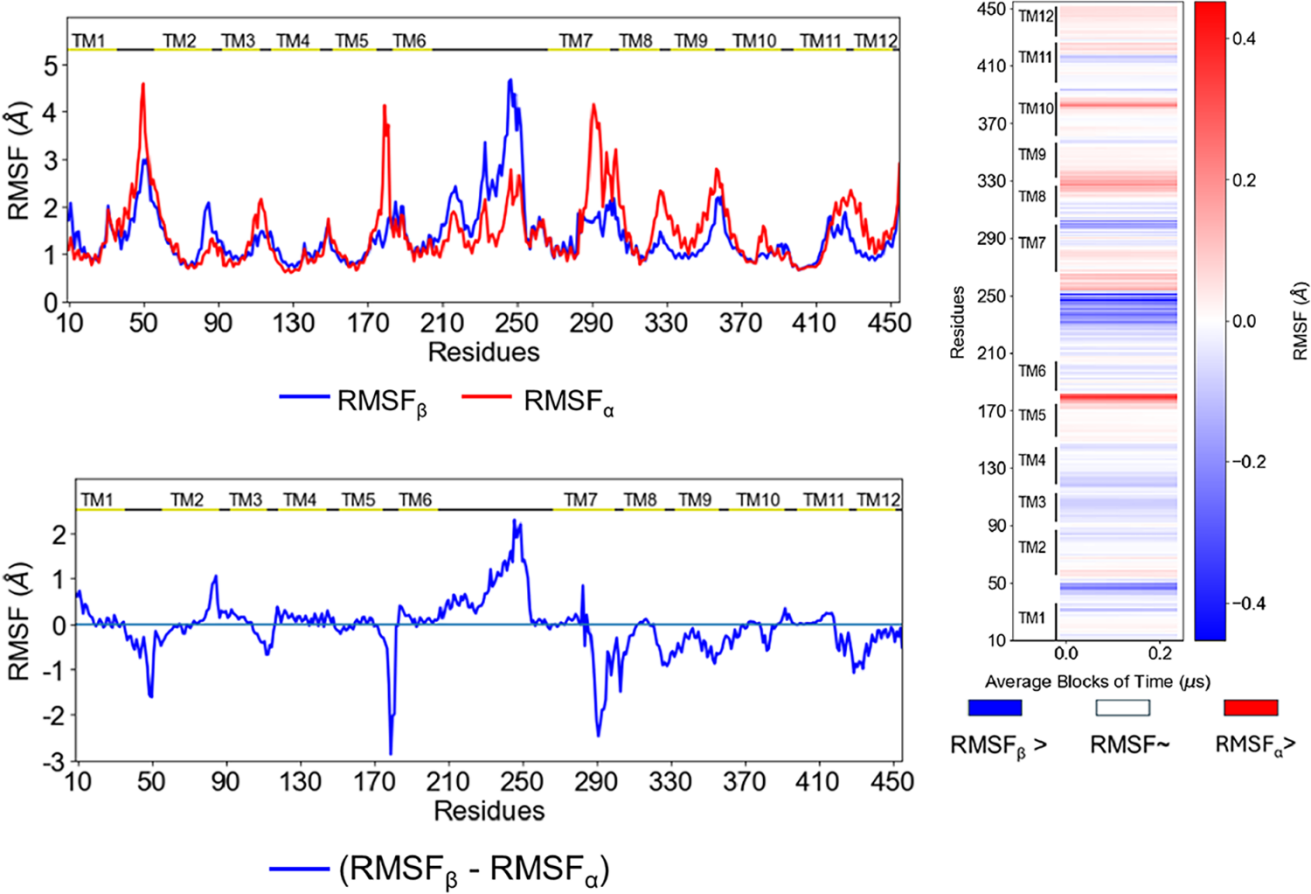
(A) Differences in RMSF for GLUT3 individual anomer simulations at 4.8 μs. Data from the simulation involving α-glucose is shown in red and in blue correspond to β-glucose. (B) The heatmap represents the difference in RMSF between α-glucose and β-glucose simulations, averaged over 250 ns intervals across 4.8 μs of simulation time. Red indicates greater RMSF in the α-glucose simulation, whereas blue indicates greater RMSF in the β-glucose simulation (i.e., RMSF_α_ – RMSF_β_ > 0 for red regions).

Notably, intracellular residues between TM6 and TM7 exhibit increased fluctuations when either alpha or beta sugar anomers enter the pore (Figure 5), demonstrating that anomer-specific effects can propagate to distal structural elements and influence the conformational dynamics of the entire transport pathway. This is seen with β-glucose as well, though to a lesser extent and earlier in the mixture simulations (Figure 5). Additionally, both anomers induce increased fluctuation of extracellular (EC) residues between TM1 and TM2 (Figure 5). Of note, there are four lysine residues in this EC region of GLUT3—Lys36, Lys39, Lys44, and Lys49—which could be of some interest despite the relatively small increases in fluctuation.

Overall, these detailed analyses demonstrate that stereoselectivity in GLUT transporters arises not only from the central high-affinity site but also from multiple peripheral and boundary regions along the pore. By combining trajectory analyses, RMSF comparisons, spatial mapping relative to the membrane, and consideration of specific structural elements, our simulations reveal a network of residues capable of sensing subtle differences between glucose anomers and transmitting these effects through both local and distal structural changes. These findings provide a more nuanced understanding of anomer-specific gating mechanisms^26^ and highlight the distributed nature of stereospecific interactions within the transporter.

## Discussion

The analysis presented here integrates MD simulations with previous crystallographic and transport studies to elucidate how glucose anomer structures influence interactions with GLUT1 and GLUT3. In aqueous solutions, β-glucose predominates at approximately 62% compared to α-glucose at 38%, with negligible open-chain forms. This asymmetric distribution results from structural differences in the orientation of the hydroxyl group at the C1 carbon. Recent *ab initio* MD simulations have confirmed that β-glucose forms the highest number of hydrophilic connections and the smallest number of hydrophobic connections to neighboring water molecules compared to other anomer conformations.^4–6^ Early studies by Faust^8^ reported that the β-anomer penetrates the erythrocyte membrane approximately three times faster than the α-anomer. Miwa et al.^14^ found approximately 1.5 times faster influx of the β-anomer but identical efflux rates for both anomers. This aligned with Barnett et al’s^7^ observation that the C-1 hydroxyl group interacts with GLUT1 hydrophobic groups on the outward-facing side of the transporter during influx, but not during efflux.^43^ In contrast, Kuchel et al.^1^ observed that α-anomer transport was approximately 1.7 times faster than β-anomer transport at 40 °C. Recent ^19^F NMR studies have demonstrated that for fluorinated glucose derivatives, GLUT1 consistently show higher specificity constants (*k*_cat_/*K*_m_) for α-anomers than β-anomer (Table 1). This corroborates the conclusion that fluorination increases the hydrophobic character of glucose derivatives, thereby increasing the transporter’s preference for α-anomers in nonaqueous, nonpolar environments.

The current MD study comparing single anomer interactions in GLUT1 and GLUT3 versus an equimolar mixed anomer population reveals several novel findings. In mixed-anomer simulations, α-glucose displaces β-glucose at external surfaces, while β-glucose preference is more predominant at the internal surface. RMSF analyses demonstrate that single anomer trajectories exhibit larger fluctuations in the extramembranous zones than mixed trajectories, with mixed trajectories reducing RMSFs by 15–30% in GLUT1 (Figure 4).

In GLUT1, the RMSFs obtained from the mixed trajectory deviate less from the single-anomer RMSFs than in GLUT3, when compared at the average of their 1 and 4.8 μs simulations, respectively (Figure 6, 1-D plots vs Figure 4, 1-D plots). These differences are particularly evident in the extracellular loops between TM1–2 and TM5–6, and in the gatekeeper residues Phe289–Tyr291 (Figures 6 vs [Fig fig4]). In simulations with α-glucose, RMSFs are higher: ∼1.5 Å on average in GLUT1 compared to ∼4.0 Å in GLUT3. In contrast, for β-glucose, the large intracellular linker between TM6–7 fluctuates within 1.5–2.5 Å in GLUT1, versus 2–4.5 Å in GLUT3 (Figure 6). Generally, in GLUT3, α-glucose RMSFs exceed those of β-glucose by 0.1–1 Å in the N-terminal half and by 1–2.5 Å in the C-terminal half. These RMSF differences correlate with anomer-specific alterations in salt-bridge stability. In GLUT1, the Arg400–Glu393 salt bridge widens channel openings in response to β-glucose hydration effects. In GLUT3, α-glucose-dependent interactions with Asp298–Lys356–Asp357 display distinct patterns, potentially facilitating opening of the outward-occluded state and access to bottleneck tyrosine residues.

The overrepresentation of α-glucose in GLUT3 crystal structures, despite its lower abundance in aqueous solution, likely results from the relatively high affinity van der Waals interactions between α-glucose and hydrophobic groups within the central channel. The effects of replacing H_2_O with D_2_O, which retards β-glucose anomerization without significantly affecting α-glucose bonding, can be explained by D_2_O’s interference with hydrophilic hydrogen bonds that predominate in β-glucose interactions. This phenomenon correlates with observations that the α-anomer is preferred at lower temperatures, while the β-anomer is favored at higher temperatures (Table 1).

Similarly, the Q_10_ between 29 and 37 °C of α- glucose at pH 7.5 is 3.6 and of β-glucose is 8.4, and of the equilibrium glucose mixture is ≈4.0, where Q_10_ describes the temperature sensitivity of a process, indicating how many times faster the transport rate becomes for a 10 °C rise in temperature.^8^ The *t*_1/2_ of equilibrated glucose penetration at 37 °C is approximately 30% faster than it would be if the anomers were transported independently, indicating that synergism between the mixed anomers increases the α-glucose penetration rate. This accords with the results of the mixed anomer trajectories showing that the presence of β-glucose permits α-glucose to access regions within GLUT1 and GLUT3 with higher frequency. The elevated Q_10_ observed for β-glucose suggests that its transport is more strongly dependent on temperature-sensitive conformational changes within the transporter. Compared with the α-anomer, β-glucose requires greater dehydration and protein rearrangement during translocation and interacts more extensively with aromatic and hydrophobic gating residues, whose side-chain motions are highly temperature dependent. In short, α transport relies less on temperature-sensitive rearrangements and more on direct recognition, resulting in a moderate *Q*_10_ of ∼3–4, whereas β transport is more strongly coupled to thermally driven gating and hydration changes, giving it a much higher Q_10_.

## Conclusions

Anomeric configuration strongly influences the way glucose interacts with its transporters; both α-glucose and β-glucose are highly polar molecules dominated by hydroxyl groups, and they engage primarily in hydrogen bonding with polar or charged amino acid residues in transporter binding pockets. The sole steric difference between the two anomers lies in the orientation of the anomeric hydroxyl group at C1: axial in the α form and equatorial in the β form. This subtle variation alters hydrogen-bond geometry and steric presentation, which can significantly affect how well a transporter stabilizes each anomer.

Structural and functional studies of facilitative (GLUT) and sodium-dependent (SGLT) glucose transporters demonstrate that the binding sites are shaped by networks of polar residues (e.g., Asn, Gln, Ser, Thr, Glu, Asp) often supported by nearby hydrophobic side chains that contribute to shape complementarity and water exclusion. Anomer selectivity arises when this polar interaction network favors the geometry of either the axial or equatorial anomeric hydroxyl. For example, intestinal SGLT1 shows higher transport rates for β-glucosides relative to their α counterparts in brush-border membrane vesicles and oocyte expression systems.^44^ Similarly, erythrocyte GLUT1 tends to transport β-glucose more efficiently, as demonstrated using nonmutarotating analogs such as C1-fluoroglucoses in NMR and kinetic assays.^45^ Structural and mutational analyses further confirm that specific residues in the binding pocket dictate anomeric preference through precise hydrogen-bonding interactions rather than broad differences in polarity.^42^

Our findings at the residue and vestibule level complement experimental observations, while also reconciling reports such as those by Carruthers and colleagues,^22^ who showed that GLUT1 transports α- and β-glucose with similar avidity in red blood cells and that biphasic transport arises from nucleotide-dependent regulation rather than anomeric preference. This highlights that subtle, anomer-sensitive interactions can exist within the transporter without necessarily producing large differences in net transport, emphasizing the mechanistic rather than purely kinetic significance of our results.

This study reveals that, contrary to previously held views based on crystallography, anomer stereospecificity in both GLUT1 and GLUT3 is not confined to the central high-affinity binding site but is instead distributed primarily within the external and internal vestibules.^11^ The differential transport of glucose anomers reflects a complex interplay between their inherent physicochemical properties and specific interactions with transporter vestibules. β-glucose’s superior hydration properties and predominance in solution correlate with its preferential interaction with internal vestibules, while α-glucose’s higher affinity for aromatic and aliphatic residues explains its preference at external vestibules. The synergy between glucose anomers, namely, the observation that α-glucose and β-glucose do not act independently but instead influence each other’s behavior in a cooperative manner, as deduced from Faust’s data, is evident in their differential hydrogen-bonding to residues in both GLUT1 and GLUT3. These effects can be explained by allosteric interactions, where the binding preferences of α-glucose and β-glucose mutually modulate transporter conformation. Faust’s experimental findings suggested that the transport of one anomer might be affected by the presence of the other, and recent simulations corroborate this by showing that α- and β-glucose form hydrogen bonds with distinct sets of residues, suggesting discrete interaction patterns. Such allosteric interplay may cause structural or dynamic changes in GLUT transporters that directly impact how each anomer is handled.

Our simulations reveal a consistent pattern of anomer-specific interactions and dynamics along the GLUT1 and GLUT3 transport pathways that closely parallels experimental observations. At the extracellular entry site, GLUT3 residues such as Arg124, Trp63, Ser64, and Gly428 show a clear α-glucose binding preference in the simulations (Table 3). This agrees with crystallographic data showing that GLUT3 structures predominantly capture α-glucose in the orthosteric site despite β-glucose being more abundant in solution.^11^

Deeper inside the transporter, both GLUT1 and GLUT3 show stronger β-glucose interactions within the intracellular vestibule (e.g., Arg212, Pro205, Asp240 in GLUT1; Gly82, Gly89, Leu213, Leu258 in GLUT3). This β-biased inner pathway is consistent with efflux experiments, where β-glucose is transported more efficiently under several net efflux conditions (Table 1). Experimental influx, efflux, and exchange assays report different α/β ratios, indicating step-specific anomer sorting along the transport cycle, precisely the pattern emerging from our residue-level interaction maps and RMSF analyses.

Simulations also show that mixed α + β occupancy produces distinct dynamic signatures: enhanced local fluctuations near α + β collision sites, redistributed H-bonding patterns, and overall lower RMSFs compared with single-anomer flooding (Figure 4 and Table 4). These effects indicate cooperative or competitive coupling between the two anomers and long-range allosteric damping. This aligns with classical kinetic studies (*e.g*., Faust^8^) reporting that α/β mixtures alter apparent *K*_m_ and *v*_max_ values relative to isolated anomers, implying multiligand allosteric modulation of the transport cycle.

Residues forming external and internal gates, particularly aromatic and charged side chains, display pronounced anomer-dependent fluctuations in our simulations. Time-resolved RMSF maps identify transient anomer-specific spikes near key salt bridges, such as Arg400–Glu393 in GLUT1 and Lys253–Glu259 or Asp298–Lys356–Asp357 in GLUT3, indicating that ligand collisions and hydration changes directly drive localized gating events (Figures 5, 6, and S4–S6). Experimentally, net flux (but not exchange) shows sharp temperature sensitivity, and β-glucose flux is more suppressed in D_2_O than α-glucose, identifying solvent-dependent gating as a β-biased control point (Table 1).^19,46^ The stronger hydration of β-glucose seen experimentally is consistent with the greater solvent-sensitive dynamics we observe.

Collectively, these results highlight a hierarchical allosteric network spanning extracellular loops, transmembrane helices, and intracellular gates in both GLUT1 and GLUT3. The observed pattern parallels established allosteric systems, such as GPCR extracellular vestibules, where ligand-induced fluctuations propagate inward to regulate orthosteric affinity,^47,48^ and calmodulin–AQP systems, where extramembranous interactions modulate gating dynamics.^49^ These findings suggest that long-range, hydration-sensitive, anomer-dependent coupling similarly influences glucose transport in GLUTs.^50^

Building on these results, future studies could explore the impact of fluorinated glucose analogues on transporter interactions, as highlighted by recent ^19^F NMR work.^51,52^ MD simulations could complement the current findings by providing mechanistic insight into how fluorination alters polarity, solvation, and hydrophobicity, and thereby affects binding dynamics and anomer selectivity. Such studies could also inform the design and interpretation of fluorinated glucose tracers for diagnostic applications, including PET imaging, by revealing how subtle chemical modifications modulate transporter kinetics and selectivity. The trends in anomeric preference identified here are based on observed interaction patterns and fluctuations in the simulations. While these results do not provide absolute binding or transport free energies, they offer meaningful atomistic insight into site-specific interactions and molecular recognition. Future studies could complement this work with free energy calculations to more rigorously quantify anomer-specific binding and transport energetics, and to explore how vestibule-based selectivity might be leveraged to modulate glucose transport in pathological conditions such as diabetes or cancer, where glucose metabolism is dysregulated.

## Supplementary Material



## Notes

Map of anomeric preferences of GLUT3 residues (Figure S1); GLUT1 and GLUT3 mixture simulations; α-/β-glucose trajectories along pore and medium (Figure S2); individual-anomer GLUT1 and GLUT3 simulations; glucose pathways; transient H-bonds; GLUT1 RMSFs (Figure S3); GLUT3 Cα RMSFs for 60 α- or β-glucose; first-microsecond RMSF differences (Figure S4); Cα displacement evolution; TM/loop annotations; RMSF comparisons at multiple intervals (Figure S5); GLUT3 individual-anomer RMSF progression (Figure S6) (PDF)
