# Supplementary material for: Atomistic Insights into Anomeric and Stereochemical Effects on Glucose Transport by GLUTs
Source: J Am Chem Soc. 2026 Feb 2;148(5):5189–201. doi: 10.1021/jacs.5c17290 (PMC12903842; doi:10.1021/jacs.5c17290)
Supplement: Supplementary file 1 [file ja5c17290_si_001.pdf]

Atomistic Insights into Anomeric and Stereochemical Effects on Glucose Transport by GLUTs

Brian Wiley,[1] Leonardo Cirqueira,[1] *Richard J. Naftalin,[2*]* *Carmen Domene[1*]*

[1]*Departments of Chemistry, University of Bath*, 1 South Building, Claverton Down, Bath BA2 7AX, United Kingdom

[2]*BHF Centre of Research Excellence, School of Medicine and Life Sciences, King's College London, Waterloo Campus Stamford St, London SE1 9HN*, United Kingdom

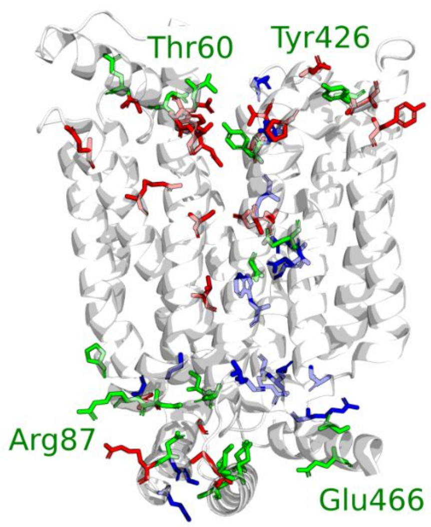

Figure 1. Map of anomeric preferences of GLUT3 residues, as presented in Table 3. Red indicates α-glucose preference, blue indicates β-glucose preference and residues with no clear preference are shown in green. Some residues are labelled for orientation and reference.

Replica	(A) GLUT1	(B) GLUT3
1	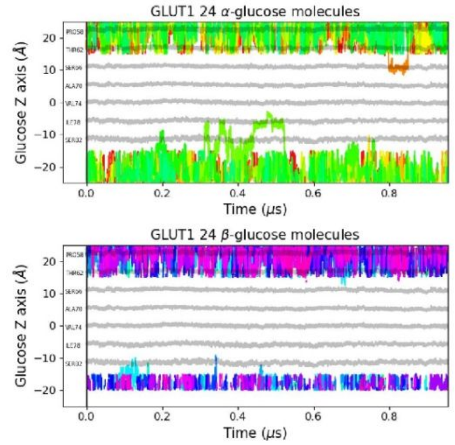	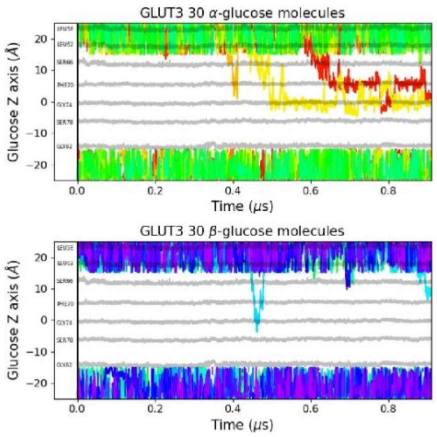
2	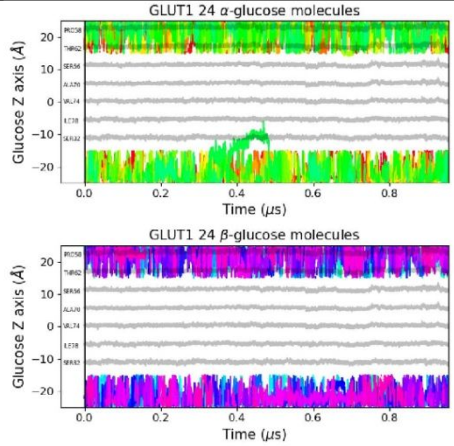	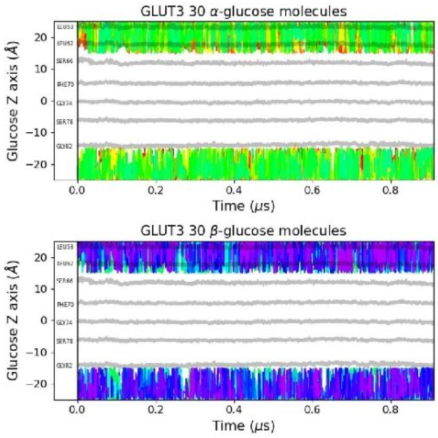
3	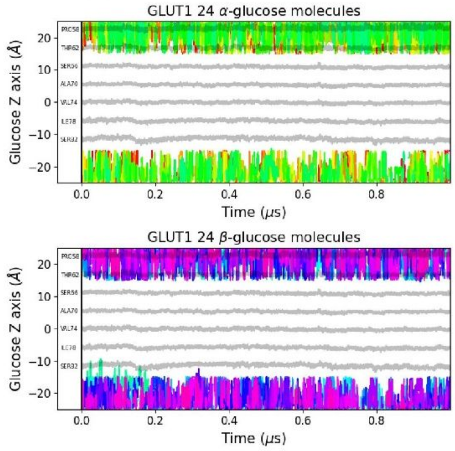	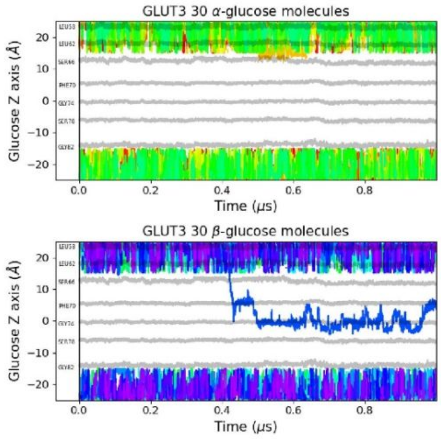

Figure S2. First microsecond of the trajectories from the simulations of GLUT1 (A) and GLUT3 (B) in the presence of α-glucose and β-glucose, as observed in the mixture simulations across three replicas. The evolution of the positions of the centre of mass of individual glucose molecules is shown, either along the main pore of the protein or in the surrounding medium, as captured in the flooding simulations. The origin of the z-axis corresponds to the centre of mass of the lipid membrane.

(A) GLUT1	(B) GLUT3
β-glucose (2 μs; 48 molecules) (> 4ns only)	α-glucose (6 μs; 60 molecules) (> 4ns only)

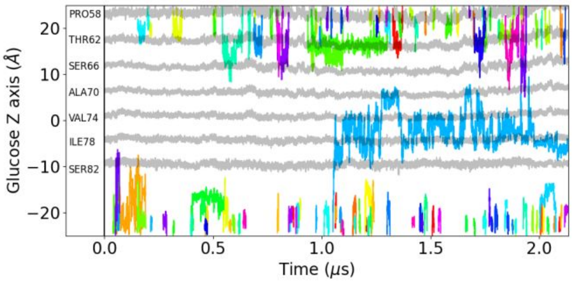

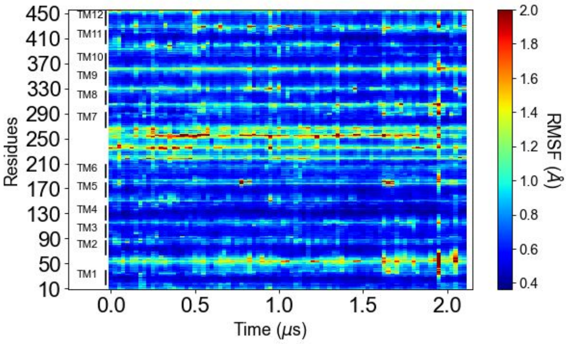

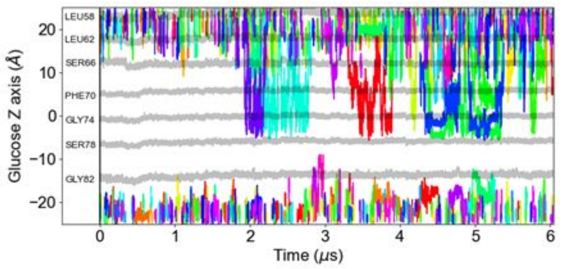

β-glucose (5 μs; 60 molecules) (> 4ns only)

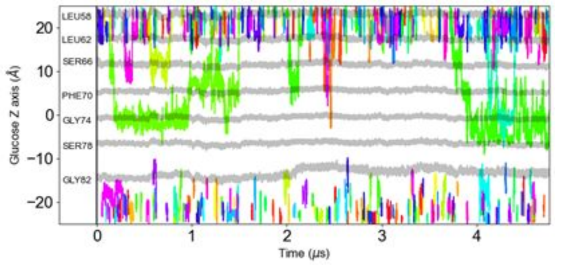

Figure S3. Simulations of (A) GLUT1 and (B) GLUT3 in the presence of α-glucose and β-glucose from the individual anomeric simulations. The pathways followed by glucose molecules through the protein pore are referenced to the crystal structures with PDB IDs 4PYP and 4ZW9, as observed in the fluid-flooded trajectories. Glucose forms multiple transient hydrogen bonds with extramembrane domains of the GLUT proteins. The evolution of the centre of mass positions of individual glucose molecules is shown, either along the main pore or in the surrounding medium. Each trace coloured differently corresponds to a glucose molecule. Only glucose molecules that remain within the pore or interact with extramembranous residues for more than 4 ns are included. The origin of the z-axis corresponds to the centre of mass of the lipid membrane. Simulations of GLUT1 also include RMSF profiles over 250-ns time periods.

(A)

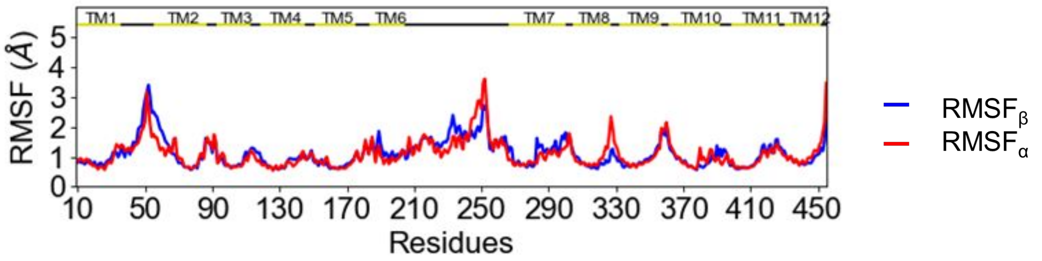

(B)

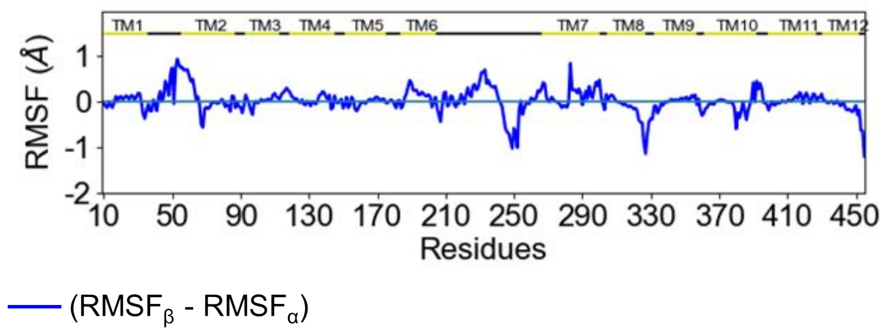

Figure S4. (A) Cα RMSFs of GLUT3 from two trajectories containing either 60 α-glucose (red) or 60 β-glucose (blue) molecules, shown at the 1-μs time point. **(B)** RMSF differences from the individual anomer simulations during the first microsecond, included for comparison with the plots in the main manuscript where the full trajectories are analyzed.

Replica	(A) GLUT1	(B) GLUT3
1	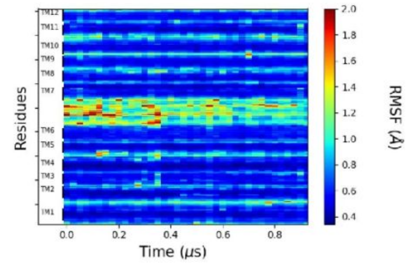	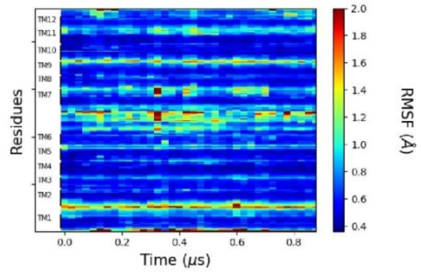
	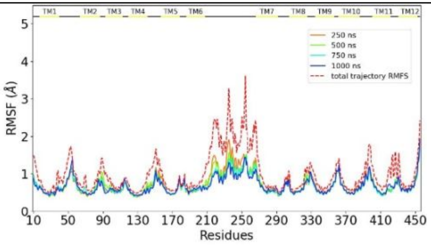	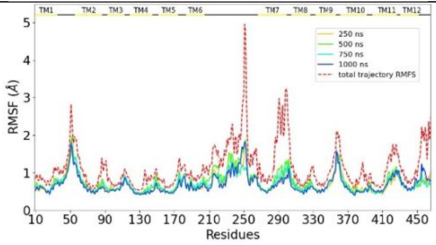
2	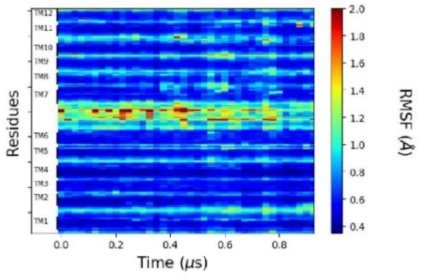	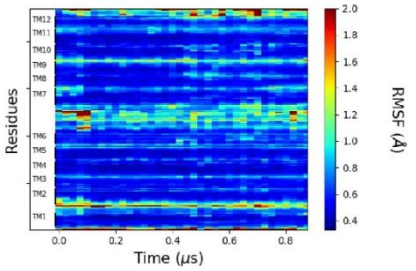
	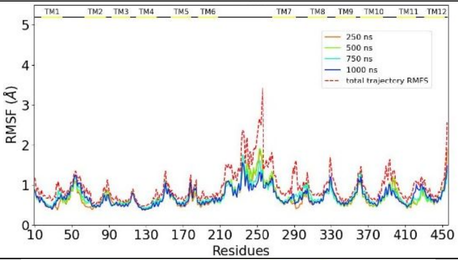	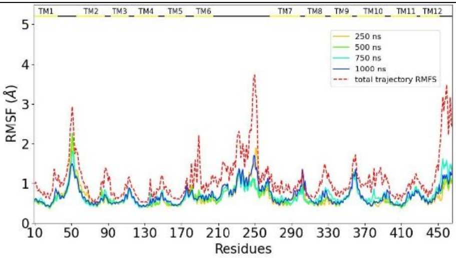
3	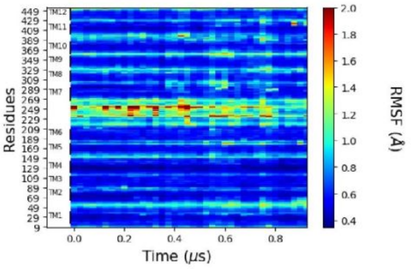	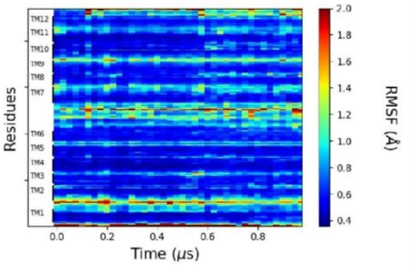
	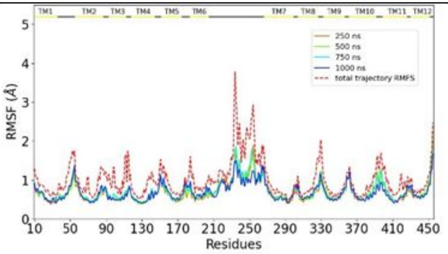	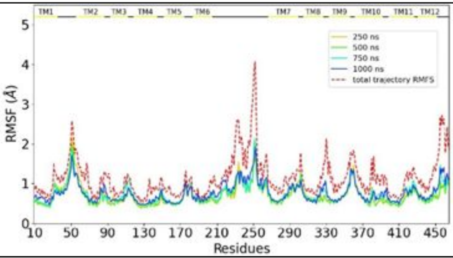

Figure S5. (Top) Time evolution of the displacement of protein Cα atoms during the trajectories, referenced to the starting structure for each of the three replicas of the mixture simulations. Residues are listed on the y-axis, along with their corresponding transmembrane (TM) or loop regions. (Bottom) Comparison of Cα RMSFs across different time intervals within the trajectories: 0.25, 0.5, 0.75, and 1 μs, as well as the full trajectory which duration is specified in Table 1. Residues (x-axis) are annotated according to their TM or loop region at the top of the figure.

α-glucose	β-glucose
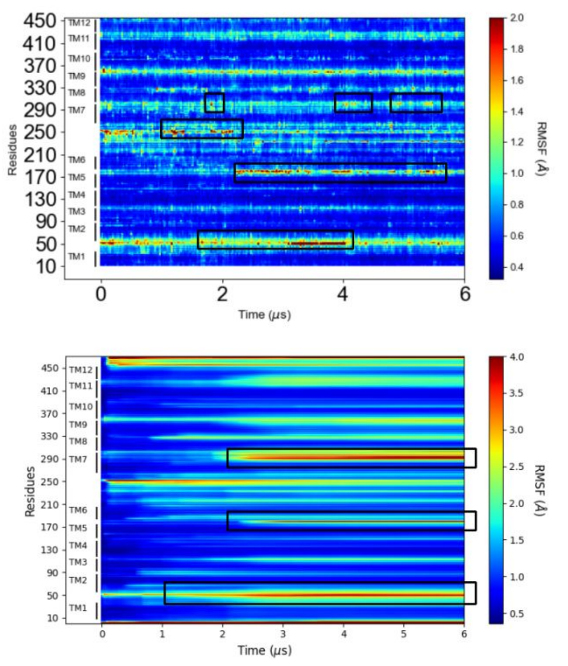	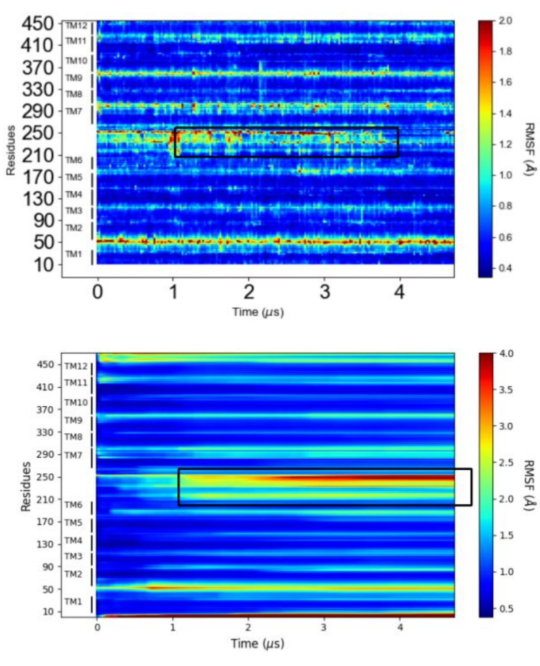

Figure S6. The progression of the RMSFs during the longer individual GLUT3 simulations with individual anomers only with 60 glucose molecules per system. Increase for α-glucose is at the vicinity of (±7 residues) Gly50, Gln170, Ser250, and Tyr290. An increase for β-glucose is only around residues Arg210 and Ser250.
